# Surgical treatment for lumbar hyperlordosis after resection of a spinal lipoma associated with spina bifida: A case report

**DOI:** 10.1097/MD.0000000000007895

**Published:** 2017-09-08

**Authors:** Tatsuya Sato, Ikuho Yonezawa, Shingo Onda, Kei Yoshikawa, Hiromitsu Takano, Yukitoshi Shimamura, Takatoshi Okuda, Kazuo Kaneko

**Affiliations:** Department of Orthopedic Surgery, Juntendo University School of Medicine, Tokyo, Japan.

**Keywords:** lumbar hyperlordosis, pediatrics, posterior fixation surgery, spina bifida, spinal lipoma

## Abstract

**Rationale:**

A hyperlordosis deformity of the lumbar spine is relatively rare, and surgical treatment has not been comprehensively addressed. In this case report, we describe the clinical presentation, surgical treatment, and medium-term follow-up of a patient presenting with a progressive lumbar hyperlordosis deformity after resection of a spinal lipoma associated with spina bifida.

**Patient concerns:**

The patient was a 20-year-old woman presenting with a progressive hyperlordosis deformity of the lumbar spine associated with significant back pain (visual analog pain score of 89/100 mm), but with no neurological symptoms.

**Diagnoses:**

The lumbar lordosis (LL), measured on standing lateral view radiographs, was 114°, with a sagittal vertical axis (SVA) of −100 mm. The patient had undergone excision of a lipoma, associated with spina bifida of the lumbar spine, at 7 months of age.

She was first evaluated at our hospital at 18 years of age for progressive spinal deformity and lumbago.

**Interventions:**

An in situ fusion, from T5 to S1, using pedicle screws with bone graft obtained from the iliac crest, was performed.

**Outcomes:**

Postoperatively, the LL decreased to 93°, and the SVA decreased to −50 mm. The decision to not correct the hyperlordosis deformity fully was intentional. Seven years and 1 month postsurgery, the patient had no limitations in standing and walking and reported a pain score of 8/100 mm; there was no evidence of a loss of correction.

**Lessons:**

Lumbar hyperlordosis after resection of a spinal lipoma associated with spina bifida is rare. Posterior fixation provided an effective treatment in this case. As the lumbar hyperlordosis deformity is often high, correction can be difficult. In this case, although the correction and fusion were performed in situ, there was no progression of either the deformity or the lumbago. Early detection remains an essential component of effective treatment, allowing correction when the spinal deformity is easily reversible.

## 1 Introduction

Most commonly, lumbar hyperlordosis develops due to spasticity and tightness in children with cerebral palsy. However, a lumbar hyperlordosis spinal deformity has also been associated with myelomeningocele, polio, congenital myopathy, trauma, dorsal rhizotomy, lumboperitoneal shunt surgery, and achondroplasia, as well as having iatrogenic and idiopathic etiologies.^[1–7]^ In cases of spina bifida, lumbar hyperlordosis is usually associated with abasia attributed to a myelomeningocele. To the best of our knowledge, surgical treatment of a lumbar hyperlordosis associated with resection of a spinal lipoma has not been previously reported. Here, we describe such a case in an ambulatory patient who developed a lumbar hyperlordosis deformity after resection of a spinal lipoma associated with spina bifida.

## 2 Case report

### 2.1 Clinical history

The patient had undergone total excision of a lipoma, associated with spina bifida, at 7 months of age. The lipoma was dissected from the dura and subcutaneous attachments. Mild lumbar hyperlordosis was noted at 13 years of age. The patient presented to our institution at 18 years of age, complaining of increasing lumbar lordosis (LL) and low back pain. At 20 years of age, she noted progression of her lumbar hyperlordosis, which was associated with increasing low back pain (visual analog scale score 89/100 mm) and difficulty maintaining a standing position. On physical examination, no neurological deficits were noted, and the patient had full range of motion of the hip joint and no gastrointestinal symptoms.

**Figure 1:**
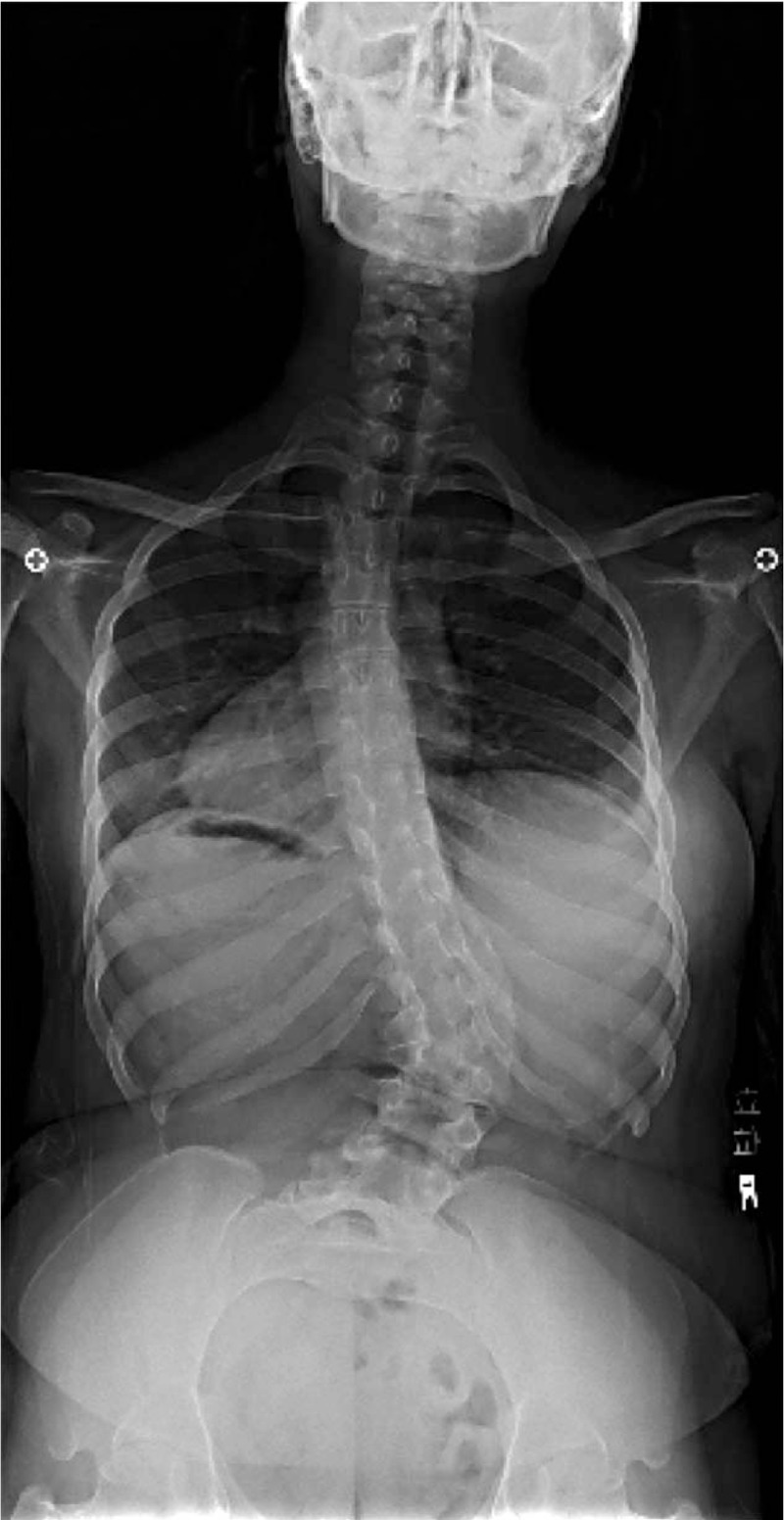
Preoperative anteroposterior radiograph showing a right lumbar curve of 41°, from T10 to L4, and a left thoracic curve of 27°, from T2 to T10.

**Figure 2:**
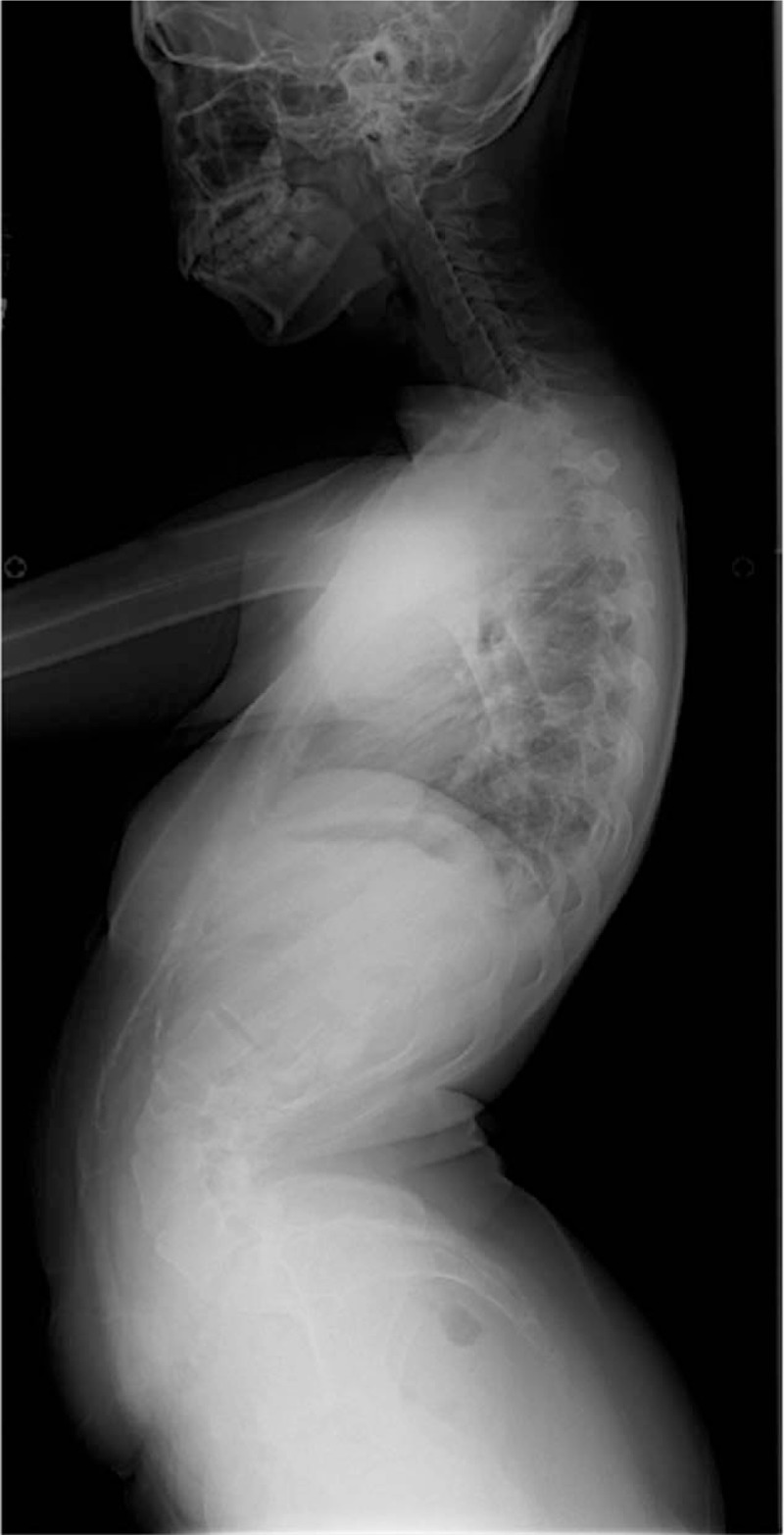
Preoperative lateral radiograph showing a hyperlordosis of 114°, from L1 to L5, and a sagittal imbalance of −100 mm.

**Figure 3:**
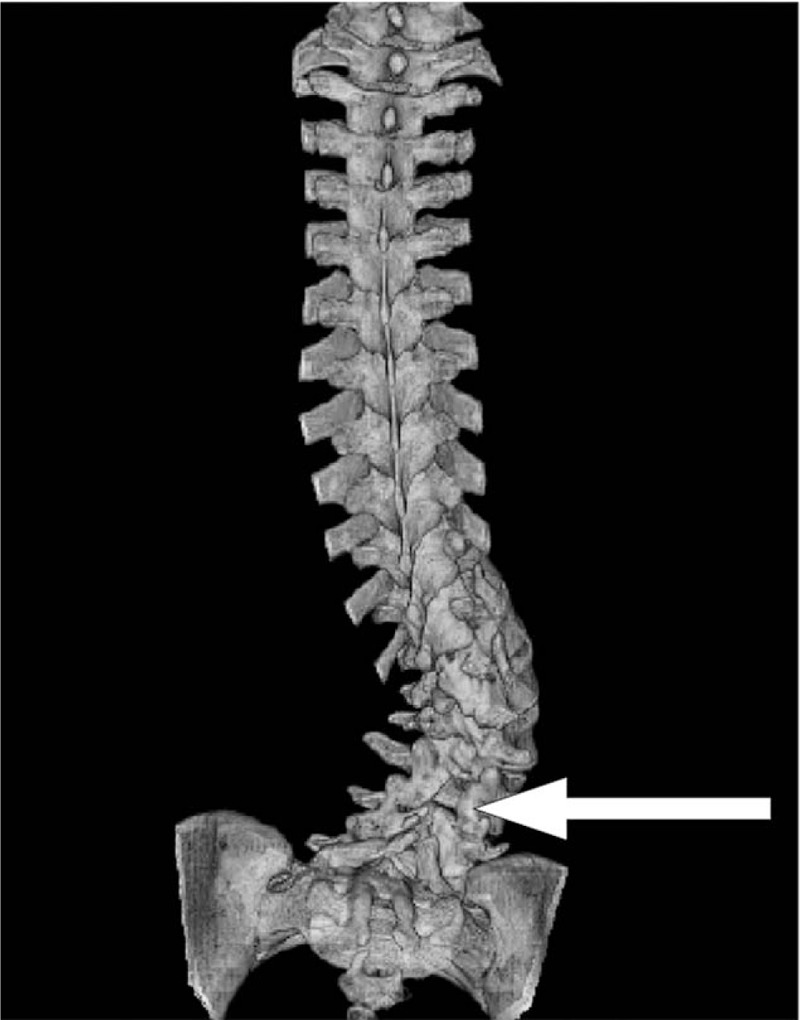
Preoperative computed tomography image showing spina bifida, from L2 to the sacrum.

**Figure 4:**
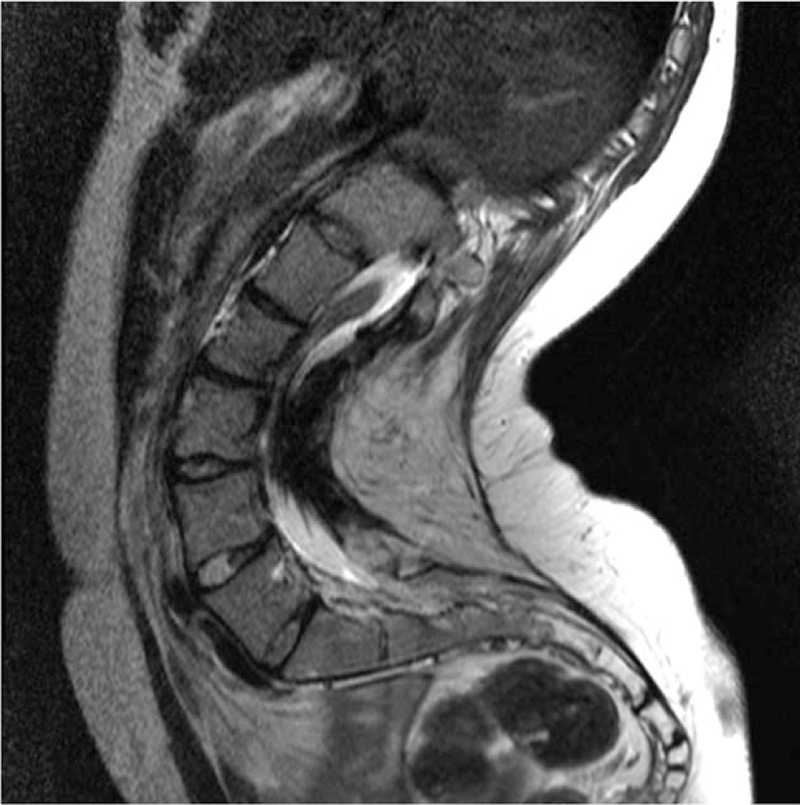
Preoperative magnetic resonance imaging showing no abnormality in the spinal canal.

Anteroposterior radiographs in standing position revealed a left thoracic curve, with a Cobb angle of 27° from T2 to T10, and a right lumbar curve of 41°, from T10 to L4. The following radiographic parameters were also measured: sacral slope (SS), pelvic tilt (PT), pelvic incidence (PI), thoracic kyphosis (TK), LL, and sagittal vertical axis (SVA). SS was defined as the angle formed by a line drawn along the sacral end plate and the horizontal plane. PT was defined as the angle formed by a line joining the middle of the sacral end plate and the hip joint axis and the vertical plane. PI was defined as the angle formed by a line drawn perpendicular to the sacral end plate and a line joining the middle of the sacral plate and the hip axis. TK was measured between the upper endplate of T5 and the lower endplate of T12, using the Cobb method. LL was measured between the upper endplate of L1 and the lower endplate of L5, using the Cobb method. Sagittal balance was measured using the SVA, which was defined as the horizontal distance of a plumb line dropped from the center of the C7 body to the posterior–superior corner of S1, with an anterior displacement of the sagittal plumb line defined as positive. Preoperative values of measured variables were as follows: LL, 114°; PT, 32°; PI, 36°; SS, 64°; TK, 50°; SVA, −100 mm; and Risser sign, 5 (Figs. 1 and 2). The computed tomography scan revealed spina bifida from L2 to the sacrum (Fig. 3), with no evidence of lesions in the spinal canal evident on magnetic resonance imaging (Fig. 4).

In situ fusion from T5 to S1 was performed using pedicle screws, inserted under fluoroscopic guidance, with bone grafting obtained from the iliac crest. Identification of the entry point for the pedicle screw was extremely difficult because of loss of the lumbosacral vertebral arch and the presence of dense adhesions from excision of the spinal lipoma. The T7 to T11 segment was fixed by sublaminar wiring, using high polymer polyethylene tape. The T5 level was selected as the upper instrumented vertebrae to prevent the development of proximal junctional kyphosis. Anterior or posterior spine release was not performed for 2 reasons. First, the hyperlordosis was primarily located in the synsacrum, with pelvic organs in close proximity, and 2nd, due to the prior lipoma extraction, the dura mater was strongly adherent to surrounding tissue, with dense scar tissue present, which made it difficult to scrape the dura mater from the surrounding tissue and increased the risk of dura mater injury.

**Figure 5:**
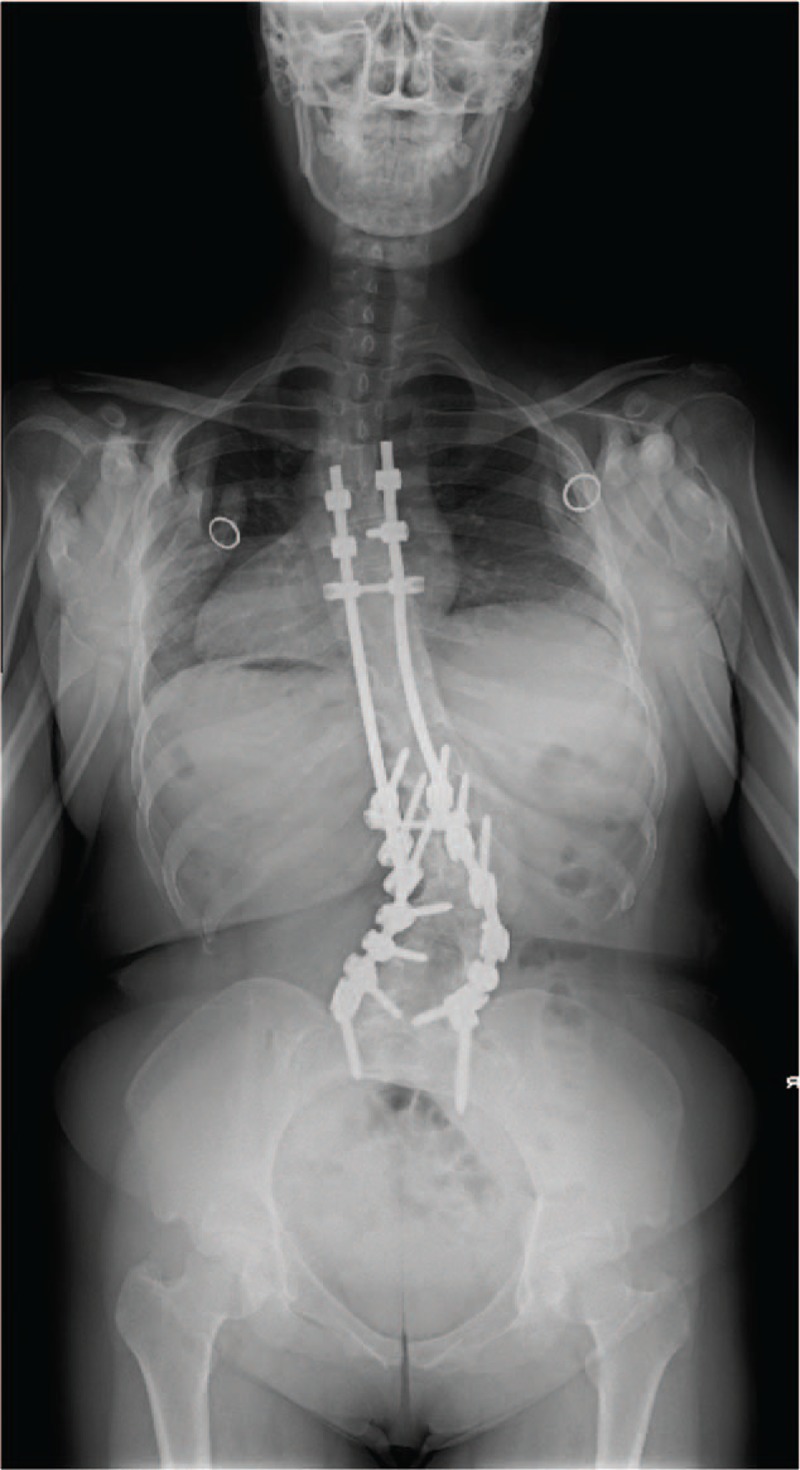
Postoperative anteroposterior radiograph showing the scoliotic lower curve corrected to 31° and upper curve corrected to 19°.

**Figure 6:**
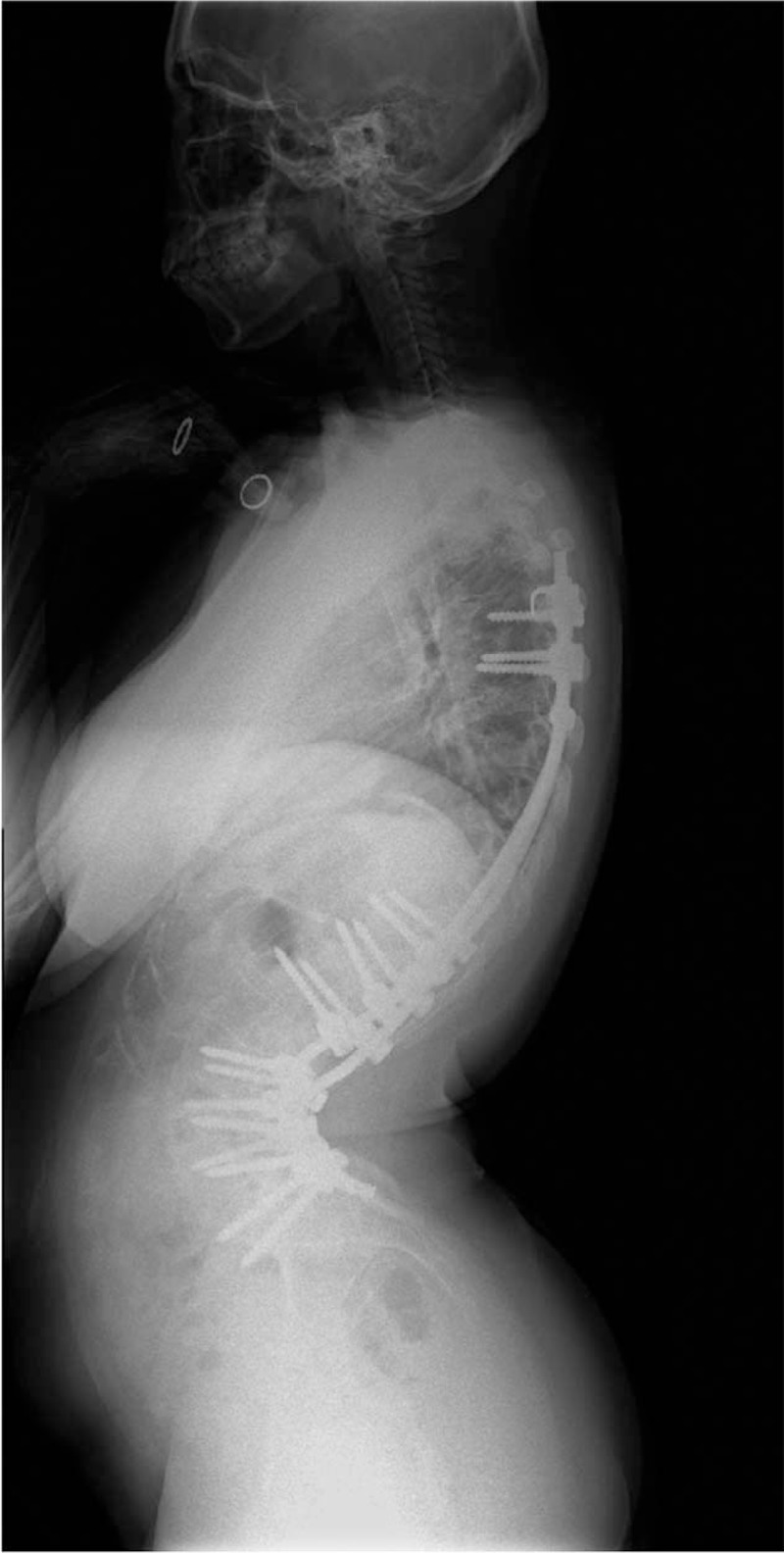
Postoperative lateral radiograph showing the hyperlordosis curve corrected to 93° and sagittal balance corrected to −50 mm.

Radiographic measurements postoperatively were as follows: the left thoracic curve from T2 to T10, Cobb angle of 19°; right lumbar curve from T10 to L4, Cobb angle of 31°; LL, 93°; PT, 21°; PI, 36°; SS, 57°; TK, 42°; and SVA, −50 mm (Figs. 5 and 6). Seven years and 1 month postsurgery, the patient had no limitations in standing and walking, and her lower back pain had improved from a visual analog scale score of 85/100 mm, preoperatively, to 8/100 mm. There was no evidence of displacement of spinal segments, breakage of the internal fixation material, or a loss of correction.

## 3 Discussion

Fifty-two percent of patients with spina bifida and a myelomeningocele have scoliosis,^[8]^ and 8% to 0% exhibit a kyphosis. By comparison, lumbar hyperlordosis is observed as a complication of spina bifida in only 1.5% of cases, even when there is a concurrent myelomeningocele.^[9]^ In children with cerebral palsy, spasticity and hypoextensibility of the iliopsoas, associated with contractures of the hip and erector spinae muscles, is a main cause of lumbar hyperlordosis.^[3,4]^ In children, strong fusion of the posterior spinal components and scarring may develop after lumboperitoneal shunt surgery, leading to the development of a lumbar hyperlordosis.^[1]^ Lumbar hyperlordosis in children can also develop as a compensation to a thoracic hyperkyphosis deformity,^[7]^ as well as due to Scheuermann disease.^[10]^ However, there is no consensus regarding the effect of thoracic hyperkyphosis on the development of lumbar hyperlordosis. In the present case, it is unclear if the posterior lumbar spinal scar tissue associated with resection of the spinal lipoma could have been the cause of the lumbar hyperlordosis deformity, or if the spina bifida was the primary cause. Although the causal relationship was unknown, there was the fact that hyperlordosis occurred after operation for spinal lipoma associated with spina bifida. Therefore, surgeons should be aware that patients with pre-existing spina bifida who undergo lipoma excision can develop a lumbar hyperlordosis deformity.

Surgical treatment is indicated when the lumbar hyperlordosis causes limitations in sitting and standing, low back pain, and eating disorders due to pressure on the digestive tract.^[4,6]^ In the present patient, surgery was performed because of the progressive nature of the lumbar hyperlordosis, which caused a severe limitation in walking and low back pain. In patients with relatively rigid moderate lumbar hyperlordosis, either reverse pedicle subtraction osteotomy^[7]^ or posterior spinal fusion after anterior release is indicated. In our patient, the lumbosacral area was principally affected by the lumbar hyperlordosis, and the approach to the synsacrum was difficult because of the proximity to the pelvic organs. Furthermore, the hyperlordosis deformity was highly advanced at the time of surgery, and, therefore, we considered it would be extremely difficult to perform discectomy using an anterior approach due to the approach angle of the surgery. Since the patient had previously undergone lipoma extraction, the presence of extensive adhesion of the dura mater with surrounding tissues, including strong scar tissue, made it difficult to perform a posterior spine release. The criteria for conservative treatment of a lumbar hyperlordosis deformity are unclear. The appropriate degree to which the deformity of lumbar hyperlordosis should be corrected is also unclear. In our case, the patient recovered her capacity for standing and walking, and her lumbago resolved after surgery. Moreover, her postoperative course was uneventful over the follow-up period of 7 years and 1 month. On the basis of these clinical outcomes, we suggest that sufficient spinal alignment of in situ fusion, as in our patient, should be one of the treatment goals. As correction of the spinal alignment is difficult in patients with a rigid lumbar hyperlordosis, it is essential to perform surgical treatment in the early stage, when the deformity is still easily reversible.

## 4 Conclusions

In our case report, we described the efficacy of posterior spinal fixation for progressive lumbar hyperlordosis following resection of a spinal lipoma associated with spina bifida. Hyperlordosis that occurs after the removal of a lipoma associated with spina bifida is a particularly difficult deformity to correct due to the extensive scar tissue, and, therefore, early detection and follow-up are essential to perform the surgical treatment in the early stage of deformity progression, when the deformity is still easily reversible.
